# Effect of regulating macrophage polarization phenotype on intervertebral disc degeneration

**DOI:** 10.1002/iid3.714

**Published:** 2022-10-27

**Authors:** Xuefeng Hou, Yucheng Shen, Minli Sun, Bing Zhang, Jiuming Dai, Dong Chen, Zhidong Liu

**Affiliations:** ^1^ Department of Orthopedics Binhai County People's Hospital Jiangsu Province China; ^2^ Department of Geriatrics Binhai County People's Hospital Binhai Jiangsu Province China

**Keywords:** inflammation, intervertebral disc degeneration, M1 polarization, macrophages

## Abstract

**Background:**

Macrophages are the only inflammatory cells that can penetrate the closed nucleus pulposus and their polarization plays an important role in intervertebral disc degeneration (IVDD). This paper attempted to investigate the pathogenesis of IVDD by altering the polarization state of macrophages.

**Methods:**

Macrophage RAW264.7 cells were induced by interferonγ (IFN‐γ) and lipopolysaccharide (LPS). The polarization of RAW264.7 cells was estimated by western blot and immunofluorescence. The expressions of inflammatory factors were detected by ELISA. Subsequently, RAW264.7 cells were treated with different concentrations of minocycline (Mino) and sinomenine (Sino), followed by the assessment of cell viability with cell counting kit‐8 kit. Then, RAW264.7 cell culture medium was collected for the culture of human nucleus pulposus cells (NPCs). Toluidine blue staining and type II collagen staining were applied to assay the level of type II collagen. The cell apoptosis, oxidative stress, and nitric oxide (NO) level were appraised by TUNEL, oxidative stress kits and NO kit, respectively. Western blot was employed to test the levels of apoptosis‐ and oxidative stress‐related proteins.

**Results:**

IFN‐γ and LPS could induce M1 polarization of RAW264.7 cells. Mino and Sino could reduce the polarization of RAW264.7 cells toward M1. M1‐polarized medium inhibited LPS‐induced activity, inflammation, and damage of NPCs, which were enhanced by Mino and Sino in medium.

**Conclusion:**

M1 polarization of macrophages promoted LPS‐induced inflammation and damage of NPCs.

## Graphical abstract

This paper attempted to investigate the pathogenesis of intervertebral disc degeneration by changing the polarization state of macrophages. We concluded that M1 polarization of macrophages promoted lipopolysaccharide‐induced inflammation and damage of nucleus pulposus cells.

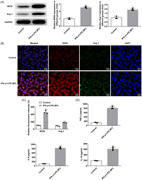

## 1 INTRODUCTION

Intervertebral disc degeneration (IVDD) is one of the main causes of low back pain and lumbar disc herniation in adults.^1^ IVDD is characterized by intervertebral disc dehydration, extracellular matrix degradation, decreased proteoglycan content and collagen type transformation, and so forth.^2^, ^3^ Once disc degeneration occurs, the structure and function of the disc cannot be maintained, thus leading to a series of problems, such as instable vertebral body, spinal canal stenosis and vertebral body deformation.^4^ The occurrence of IVDD has seriously affected the quality of human life.

A case of previous study has shown that macrophages are the only inflammatory cells that can penetrate the closed nucleus pulposus, and its number is positively correlated with the degree of IVDD,^5^ suggesting that macrophages may play a key role in the development of IVDD. In addition, as an important component of immune response, macrophages are widely involved in pro‐ and anti‐inflammatory processes as well as tissue remodeling through phenotypic transformation.^6^ M1 macrophages and M2 macrophages have been evidenced to be associated with the progression of IVDD and are highly correlated with the degree of IVDD.^7^ The M2 polarization induced by epigenetic regulation and the inhibition of transforming growth factor‐beta1 (TGF‐β1) expression that released from polarized M2 macrophages can significantly reduce the apoptosis of IVDD area, which may have significant therapeutic effects on lumbar disc degeneration.^8^ Magnoflorine alleviates M1 polarized macrophage‐induced IVDD through repressing the HMGB1/Myd88/NF‐κB pathway and NLRP3 inflammasome.^9^ Furthermore, the number of M1 macrophages increases with the aggravation of IVDD disease, while that of M2 cells has no significant change.^10^ The above research made us wonder whether IVDD disease could be ameliorated by artificially altering the transformation of M1 macrophages into M2 macrophages.

Therefore, in this paper, the effect and mechanisms of artificial regulation of macrophage polarization status were discussed in an in vitro cell model of IVDD. Our study provides a theoretical basis for the clinical treatment of IVDD.

## 2 MATERIALS AND METHODS

### 2.1 Cell culture and treatment

Mouse macrophage RAW264.7 cells that obtained from BeNa Culture Collection and primary human nucleus pulposus cells (NPCs, cat. no. CSI113Hu01) that obtained from Uscn Life Science Inc. were cultured in DMEM with addition of 10% FBS and 1% penicillin/streptomycin (all from Gibco) at 37°C with 5% CO_2_. To induce the polarization of macrophages, RAW264.7 cells were treated with interferonγ (IFN‐γ) (20 ng/ml; Sigma‐Aldrich) and lipopolysaccharide (LPS; 100ng/ml; Sigma‐Aldrich).^11^ The cells in Control group were not given any special treatment. Minocycline (Mino, 10, 20, 50, 100, 200 μM; Sigma‐Aldrich)^12^ and sinomenine (Sino, 5, 10, 25, 50, 100 μg/ml; Sigma‐Aldrich)^13^ were applied to administrate macrophages for 24 h based on the successful induction of polarization. Subsequently, the culture solution was replaced with normal medium, and then RAW264.7 cells were cultured for 48 h in each group. Conditioned medium was collected for culturing NPCs, and NPCs were divided into five groups: Control, LPS, LPS + M1 medium, LPS + M1/Mino medium and LPS + M1/Sino medium.

### 2.2 Western blot

Cells were lysed with RIPA buffer (Sigma‐Aldrich) to obtain total proteins, and a BCA Kit (Thermo) was utilized for the quantification of these protein contents. Proteins were separated by sodium dodecyl sulphate‐polyacrylamide gel electrophoresis and then were transferred to PVDF membranes (All from Sigma‐Aldrich). After the overnight cultivation with specific primary antibodies at 4°C, the membranes were incubated with secondary antibodies (1:5000; ab150113; Abcam). The protein bands were visualized with enhanced chemiluminescence reagents and then analyzed with the ChemiDoc XRS system (Bio‐Rad). The following were the primary antibodies: inducible nitric oxide synthase (iNOS) (1:1000; ab178945; Abcam), arginase‐1 (Arg‐1) (1:1000; ab124917; Abcam), Bax (1:1000; ab32503; Abcam), B‐cell lymphoma‐2 (Bcl‐2,1:1000; ab182858; Abcam), Caspase3 (1:1000; ab184787; Abcam), pro‐Caspase3 (1:1000; ab32499; Abcam), matrix metalloproteinase 2 (MMP2), 1:1000; ab92536; Abcam), MMP9 (1:1000; ab76003; Abcam) and GAPDH (1:1000; ab8245; Abcam).

### 2.3 Immunofluorescence (IF)

RAW264.7 cells were fixed with 4% paraformaldehyde for 20 min at room temperature. Then, cells were blocked with 5% goat serum for 1 h and incubated with primary antibodies against iNOS (1:80; ab170941; Abcam) and Arg‐1 (1:100; ab170941; Abcam) overnight at 4°C. On the next day, the membranes were cultivated with goat anti‐rabbit secondary antibody (1:100; ab6721; Abcam) for 1 h at room temperature. diamidino‐phenyl‐indole (DAPI) staining was utilized for cell dyeing for 5 min in the dark. The cells were photographed under a fluorescence microscope after blocking with anti‐fade mounting medium. The fluorescence intensity was observed using Image J software (version 1.46; National Institutes of Health).

For type II collagen, RAW264.7 cells were fixed with 4% paraformaldehyde for 20 min at room temperature. Then, cells that blocked with 5% goat serum for 1 h were incubated with primary antibody against Collagen II (1:100; ab34712 Abcam) overnight at 4°C, after which was the cultivation with a fluorescent goat anti‐rabbit IgG H&L (Alexa Fluor® 488) secondary antibody (1:100; ab150077 Abcam). DAPI staining was utilized for cell dyeing for 5 min in the dark. The fluorescence was observed using a laser scanning confocal microscope (magnification; x200; Olympus Corporation). The fluorescence intensity was observed using Image J software (version 1.46; National Institutes of Health).

### 2.4 Enzyme‐linked immunosorbent (ELISA)

The supernatant levels of tumor necrosis factor‐α (TNF‐α), interleukin‐6 (IL‐6) and interleukin‐10 (IL‐10) were measured using quantitative ELISA kits (R&D Systems) in line with the producer's instructions. The results were expressed in pg/ml. The level of nitric oxide (NO) was detected by NO assay kit (Nanjing Jiancheng Biotech) strictly in accordance with the manufacturer's standard procedures.

### 2.5 Cell counting kit (CCK)−8

After the coinduction with IFN‐γ and LPS, RAW264.7 cells were administrated with Mino and Sino for 24 h. Then, 10 μl of CCK‐8 solution (Dojindo Molecular Technologies) was added into each well and optical density  value was detected at 450 nm with a microplate reader (Bio‐Rad Laboratories).

### 2.6 Toluidine blue staining

The NPCs were fixed in 4% paraformaldehyde for 20 min, and then stained with 1% toluidine blue dye solution (Sangon Biotech) for 30 min at room temperature. The content of Aggrecan was observed using a fluorescence microscope (Keyence).

### 2.7 TUNEL

A TUNEL staining kit (Beyotime) was used to detect the level of apoptosis following the recommendations of manufacturer. The level of apoptosis was determined by green fluorescence in the nucleus. Five randomly selected fields were observed under a fluorescence microscope (Olympus).

### 2.8 Oxidative stress index detection

The levels of reactive oxygen species (ROS), superoxide dismutase (SOD) and malonaldehyde (MDA) were measured by ROS assay kit, SOD assay kit and MDA assay kit (Nanjing Jiancheng Biotech) as per the operations of supplier.

### 2.9 Statistical analysis

GraphPad Prism 8.0 software (GraphPad Software Inc.) was used for statistical analysis. All data were presented as the mean ± standard deviation. Unpaired Student's *t* test was utilized to demonstrate the statistical differences between two groups. Differences among multiple groups were analyzed with one‐way analysis of variance, followed by Tukey's post hoc test. A value of *p* less than .05 was regarded to be statistically significant. All experiments are statistically significant, that is, the sample size is greater than or equal to 3.

## 3 RESULTS

### 3.1 Induction of polarization of macrophages

**Figure 1:**
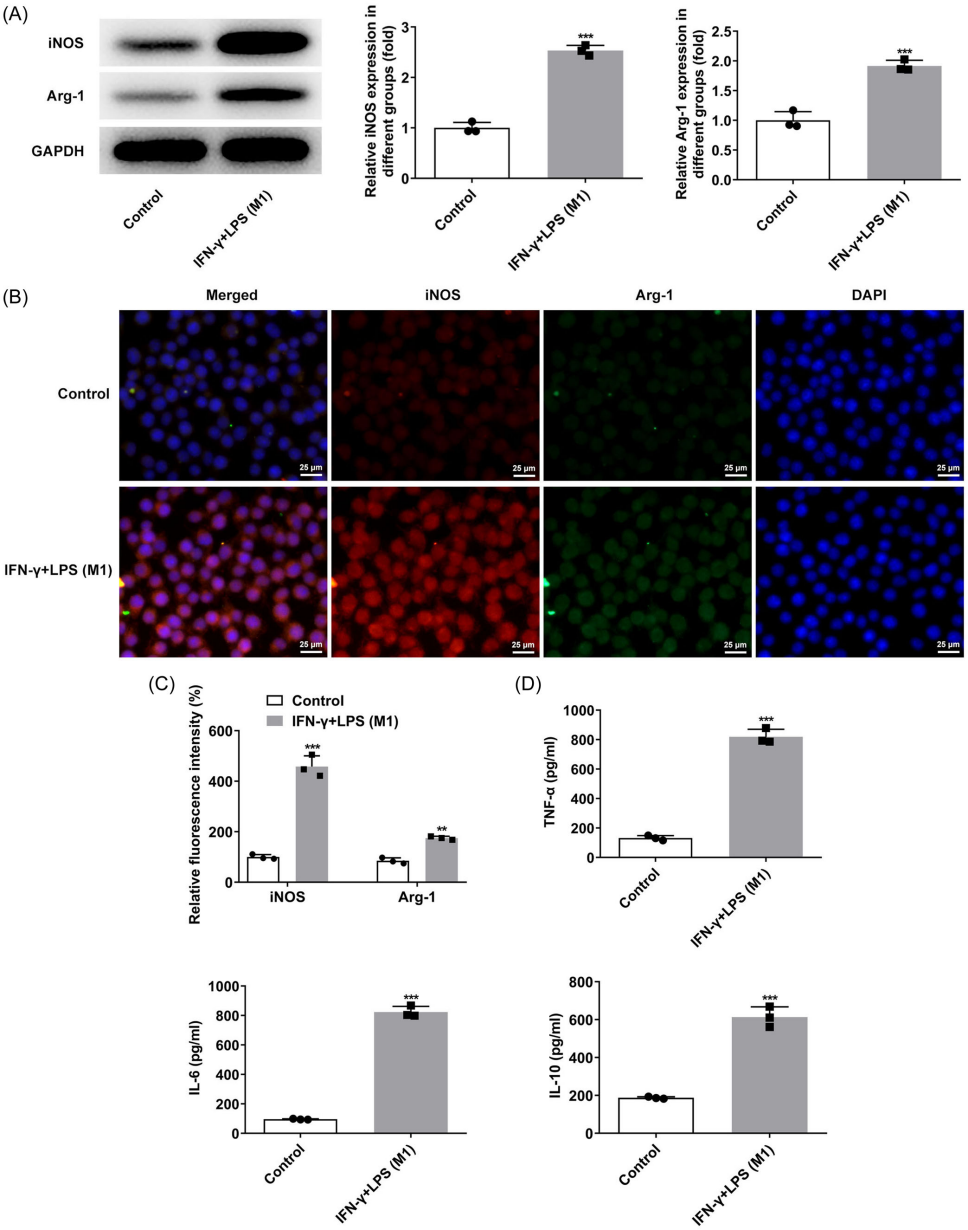
Induction of polarization of macrophages. (A) Western blot detected the expressions of iNOS and Arg‐1. (B and C) IF detected the expressions of iNOS and Arg‐1. (D) ELISA was used to detect the expressions of TNF‐α, IL‐6 and IL‐10. ***p < .001 versus Control. Arg‐1, arginase‐1; IF, Immunofluorescence; IL‐6, interleukin‐6; IL‐10, interleukin‐10; iNOS, inducible nitric oxide synthase; TNF‐α, tumor necrosis factor‐α.

After IFN‐γ and LPS were used to induce RAW264.7 cells, the expressions of iNOS and Arg‐1 were detected by western blot and IF. Compared with the Control group, iNOS expression was remarkably increased while Arg‐1 expression was slightly increased (Figure 1A,B,C). It was also discovered that the M1/M2 ratio of cells was increased after IFN‐γ + LPS induction. Afterwards, ELISA results showed that the levels of TNF‐α, IL‐6, and IL‐10 were markedly increased in IFN‐γ + LPS group compared with the Control group (Figure 1D). The above results illustrated that the polarization state of M1 macrophages was successfully induced.

### 3.2 Mino and Sino inhibited macrophage transformation to M1 type

**Figure 2:**
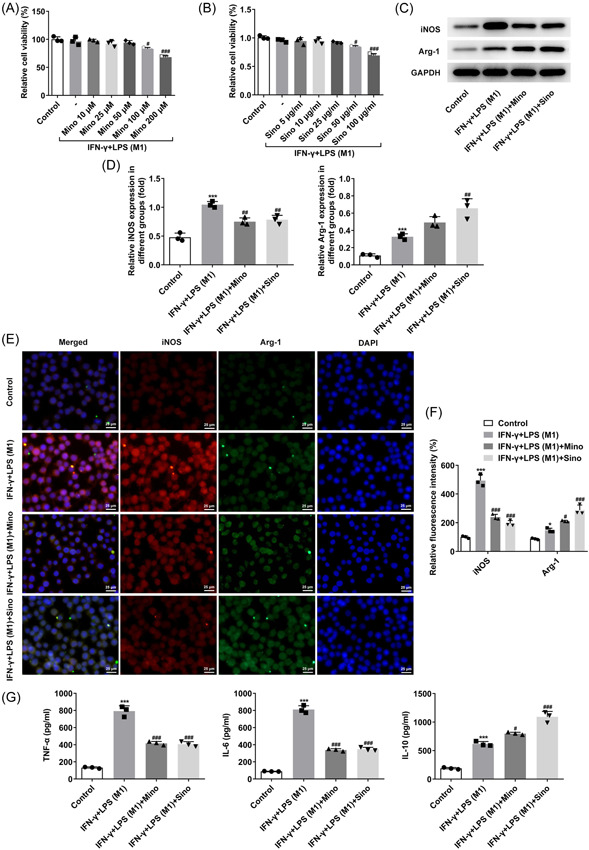
Mino and Sino inhibited macrophage transformation to M1 type. (A and B) CCK‐8 detected the cell viability. ^#^ *p* < .05, ^###^ *p* < .001 versus IFN‐γ + LPS (M1). (C and D) Western blot detected the expressions of iNOS and Arg‐1. (E and F) IF detected the expressions of iNOS and Arg‐1. (G) ELISA was used to detect the expressions of TNF‐α, IL‐6 and IL‐10. *p < .05, ***p < .001 versus Control; ^#^ *p* < .05, ^##^ *p* < .01, ^###^ *p* < .001 versus IFN‐γ + LPS (M1). Arg‐1, arginase‐1; CCK‐8, cell counting kit; IF, Immunofluorescence; IFN‐γ, interferonγ; IL‐6, interleukin‐6; IL‐10, interleukin‐10; iNOS, inducible nitric oxide synthase; TNF‐α, tumor necrosis factor‐α.

Different concentrations of Mino and Sino were applied to treat RAW264.8 cells induced by IFN‐γ and LPS. Subsequently, results obtained from CCK‐8 demonstrated that compared with IFN‐γ + LPS group, Mino (50 μM) and Sino (25 μg/ml) significantly changed the cell viability, so these two concentrations were selected for subsequent experimental studies (Figure 2A,B). The cells were then divided into four groups: Control, IFN‐γ + LPS, IFN‐γ + LPS + Mino, and IFN‐γ + LPS + Sino. Compared with the IFN‐γ + LPS group, iNOS expression was significantly inhibited while Arg‐1 expression was notably increased after the administration with Mino and Sino (Figure 2C−F), implying that Mino and Sino promoted the change of cell polarization from M1 to M2. ELISA results showed that compared with IFN‐γ + LPS group, TNF‐α and IL‐6 expressions were largely inhibited while IL‐10 expression was further increased after Mino and Sino administration (Figure 2G).

### 3.3 M1 macrophages promoted the expression of type II collagen and Aggrecan in LPS‐induced myeloid cells

**Figure 3:**
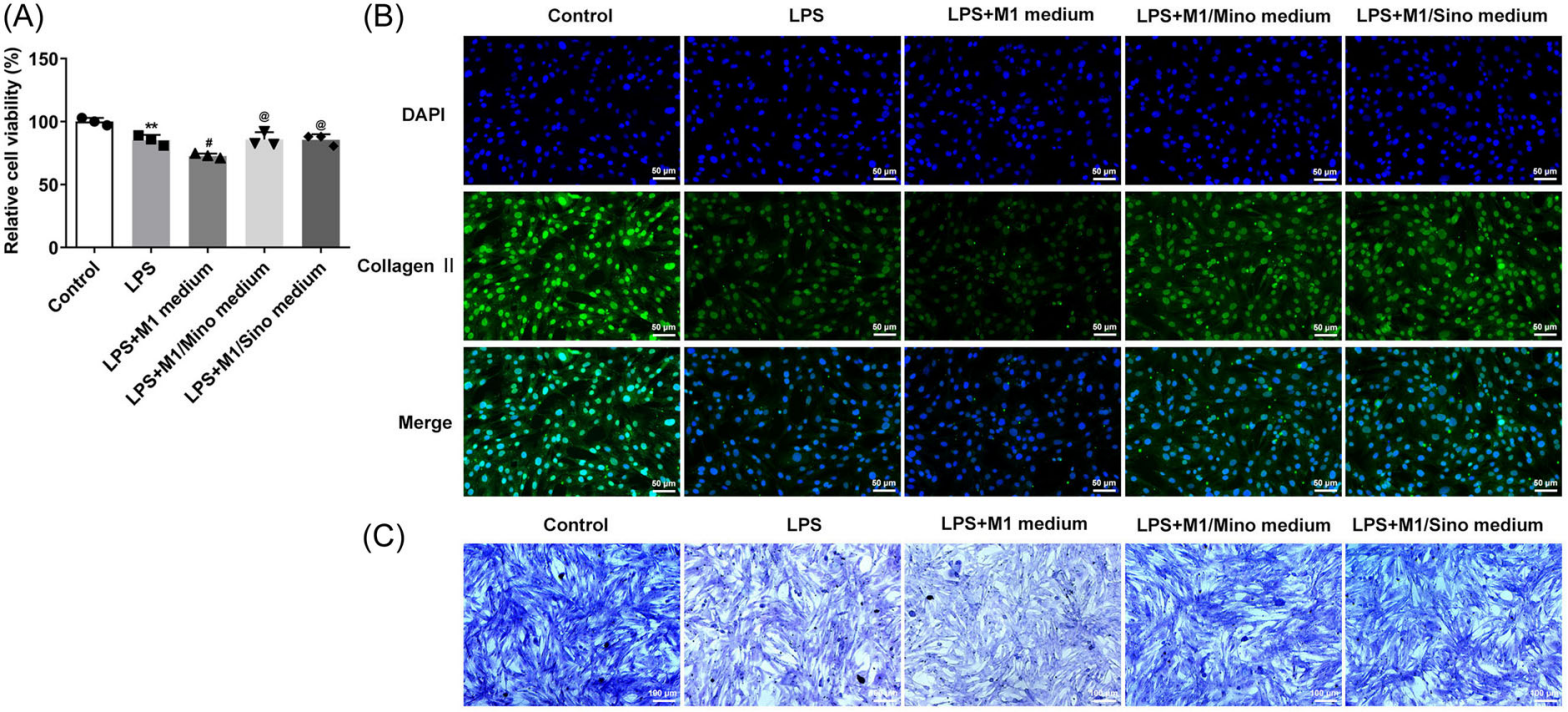
M1 macrophages promoted the expressions of type II collagen and Aggrecan in LPS‐induced myeloid cells. (A) CCK‐8 detected the cell viability. Type II collagen staining (B) and toluidine blue staining (C) detected the secretion levels of Type II collagen. *p < .05 versus control; ^#^ *p* < .05 versus LPS. CCK‐8, cell counting kit; LPS, lipopolysaccharide.

The culture medium of NPCs was replaced by the above macrophage culture medium and was used to incubate the cells for 48 h. It can be seen that LPS induction decreased the cell viability compared with control group, after further addition of M1 medium, cell viability was decreased again. Compared with that in LPS + M1 medium, cell viability in M1/Mino medium and M1/Sino medium showed an increasing trend (Figure 3A). Additionally, the expressions of type II collagen and Aggrecan were decreased in cells after LPS induction compared with the Control group, which were further reduced in LPS + M1 medium group relative to the LPS group. The decreased expressions of type II collagen and Aggrecan in LPS + M1 medium group were increased in the LPS + M1/Mino medium and LPS + M1/Sino medium groups (Figure 3B,C).

### 3.4 M1‐type macrophages promoted LPS‐induced apoptosis of myeloid cells and oxidative stress injury

**Figure 4:**
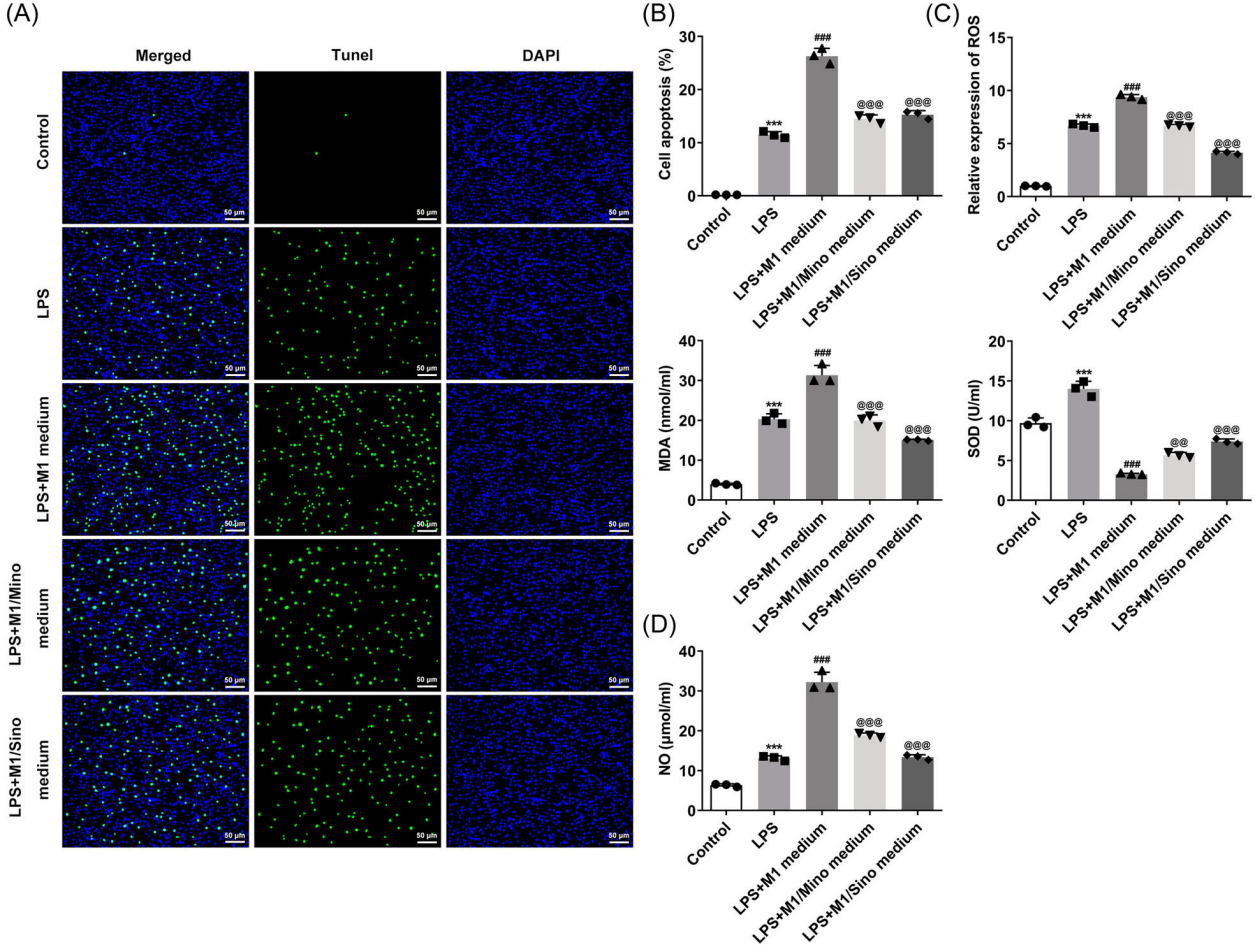
M1‐type macrophages promoted LPS‐induced apoptosis of myeloid cells and oxidative stress injury. (A and B) TUNEL assay detected the apoptosis. (C) The expressions of ROS, MDA and SOD were detected by the corresponding kits. (D) NO detection kit detected the level of NO. ***p < .001 versus Control; ^###^ *p* < .001 versus LPS; p@@@ < .001 versus LPS + M1 medium. LPS, lipopolysaccharide; MDA, malonaldehyde; NO, nitric oxide;  ROS, reactive oxygen species; SOD, superoxide dismutase.

**Figure 5:**
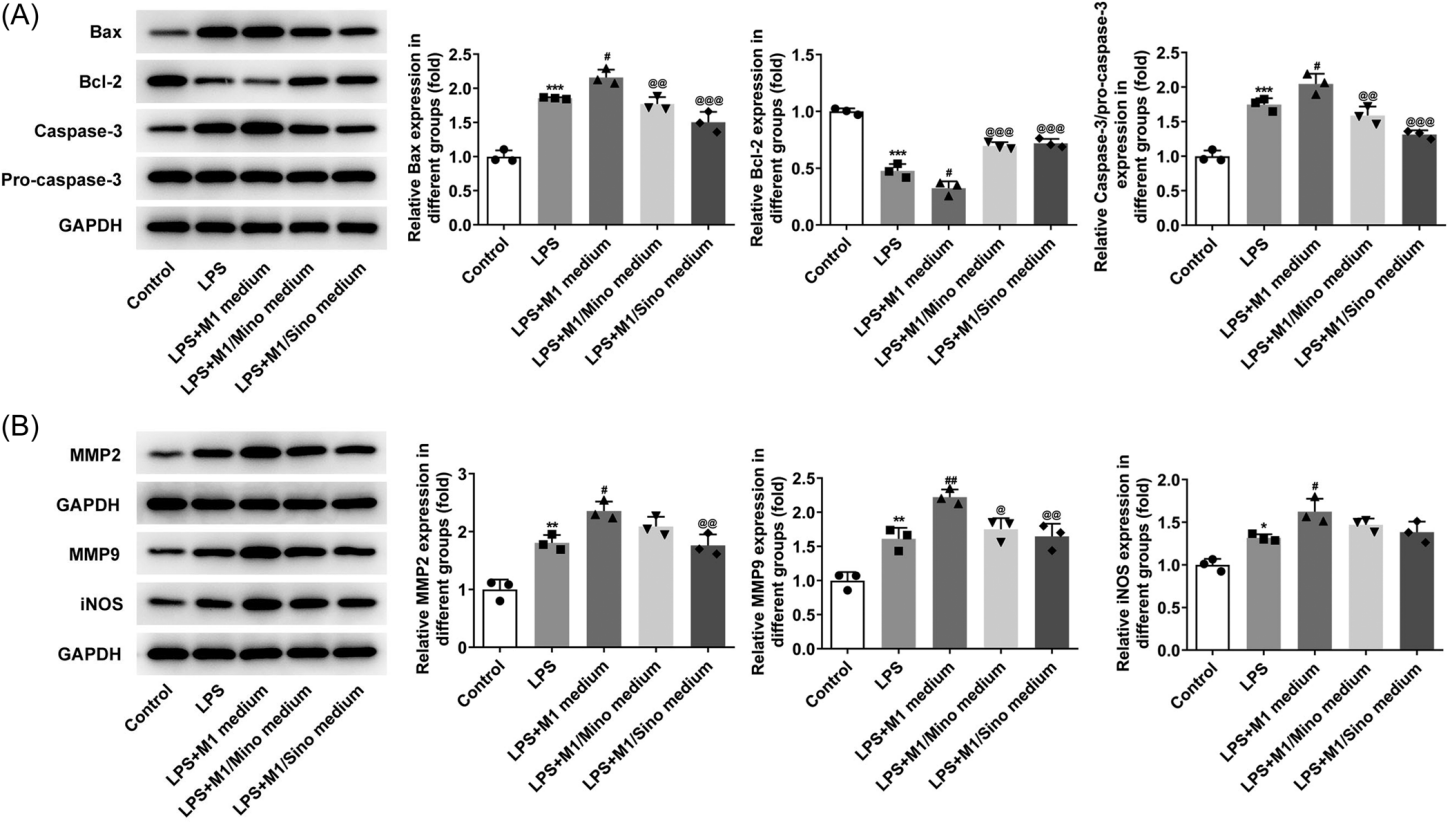
Expressions of related proteins. (A) Western blot detected the expressions of Bax, Bcl‐2 and caspase‐3. (B) Western blot detected the expressions of MMP2, MMP9 and iNOS. *p < .05, **p < .01, ***p < .001 versus Control; ^#^ *p* < .05, ^##^ *p* < .01 versus LPS; ^@^ *p* < .05, p@@ < .01, p@@@ < .001 versus LPS+ M1 medium. iNOS, inducible nitric oxide synthase; LPS, lipopolysaccharide.

Next, TUNEL results showed that the apoptosis level was remarkably enhanced after LPS induction compared with the Control group, and was further increased in LPS + M1 group. It was also discovered that the increased apoptosis level was significantly suppressed in the LPS + M1/Mino medium and LPS + M1/Sino medium groups relative to the LPS + M1 group (Figure 4A,B). Subsequently, it can be clearly observed that after LPS induction, the expressions of ROS and MDA in cells were significantly elevated while SOD expression was decreased. Compared with LPS group, the expressions of ROS and MDA in LPS + M1 group were further increased and SOD expression was further decreased. However, the expressions of ROS, MDA and SOD were all reversed after the administration with Mino and Sino (Figure 4C). The changes of NO level in each group were consistent with ROS (Figure 4D). Besides, results obtained from western blot demonstrated the upregulated expressions of Bax, Caspase‐3, MMP2, MMP9, and iNOS as well as downregulated expression of Bcl‐2 in LPS‐induced cells compared with the Control group. By contrast to the LPS group, the expressions of Bax, Caspase‐3, MMP2, MMP9, and iNOS in LPS + M1 group were further increased, while the expression of Bcl‐2 was further decreased. Compared with the LPS + M1 group, the expressions of above proteins were significantly reversed in LPS + M1/Mino medium and LPS + M1/Sino medium groups (Figure 5A,B).

## 4 DISCUSSION

IVDD is the result of intervertebral disc NPCs senescence, apoptosis, loss of cell viability and reduced synthesis of extracellular of matrix.^14^ Several studies have deeply investigated the mechanism of IVDD by regulating NPCs in vitro.^15^, ^16^ Likewise, in this paper, the mechanism of IVDD was discussed by regulating LPS‐induced NPCs in vitro.

Research has shown that macrophages are the only inflammatory cells that can get into the disc.^7^ Macrophages were found in large numbers in the discs of patients with IVDD. Macrophages can polarize into M1 and M2 types, and M1‐type macrophages can secrete a variety of proinflammatory cytokines, such as IL‐6 and TNF‐α.^17^ The levels of these proinflammatory factors secreted by cells were found to be markedly elevated in IVDD,^4^ which promoted the degradation of extracellular matrix, facilitated the phenotype change of cells and resulted in an imbalance of catabolism and anabolism, thus leading to the occurrence of IVDD.^18^ M2a Macrophage‐secreted CHI3L1 promoted the extracellular matrix metabolic imbalances via the activation of IL‐13Rα2/MAPK pathway in rat IVDD.^19^ The polarity of macrophages around newly developed microvasculature might be altered by cervical IVDD. Abundant infiltrating M1 macrophages around the vessels were associated with chronic inflammation; however, their number got suppressed following the infiltration of M2 macrophages.^20^ In terms of the disease mechanism of IVDD at the cellular level, the role that macrophages play in NPCs, and the impacts of macrophages on cell status and function were unclear. Therefore, this paper focused on the regulation of M1‐type polarization of macrophages on IVDD.

In the experiments, the polarization of RAW264.7 cells was induced by IFN‐γ and LPS. It was found that the expressions of M1 indicator iNOS and M2 indicator Arg‐1 were significantly increased in RAW264.7 cells induced by IFN‐γ and LPS, but the change in Arg‐1 was not as obvious as that in iNOS. In addition, the expressions of TNF‐α and IL‐6 in M1‐type polarization of macrophages were significantly increased. These results indicated that the coinduction of IFN‐γ and LPS could significantly induce M1‐type polarization of macrophages. Mino is a tetracycline antibiotic with a variety of nonantibacterial uses and a significant inhibitory effect on the production of inflammatory mediators and matrix metalloproteinases.^21^ Mino also regulated the polarization type of macrophages. Previous investigation has proved that Mino regulated the polarization state of macrophages to mediate neuronal inflammation and reduce peripheral nerve adhesion.^22^ On the other hand, Sino is the dry vine stem of the plant Sino and Sino. Sino has been reported to promote the polarization of macrophages to M2 phenotype.^23^ Therefore, in our experiment, macrophages were treated with Mino and Sino respectively to change the polarization state of macrophages. After selecting the concentration of Mino and Sino that had no damage to cells, these two concentrations were applied to treat RAW264.7 cells coinduced by IFN‐γ and LPS. Subsequently, it was clearly observed that RAW264.7 cells transformed from M1 to M2.

In general, induction of cells in vitro with conventional doses of LPS alone has relatively little effect on cell viability itself (the proliferation of some cells was actually promoted). However, the addition of macrophage conditioned medium significantly expanded the damage effect. In view of this, we added macrophage conditioned culture medium to explore the effect of macrophage polarization state on NPCs in vitro. We found that macrophage medium polarized to M1‐type significantly inhibited LPS‐induced NPCs viability, promoted apoptosis, increased oxidative stress injury, and downregulated the expressions of type II collagen and Aggrecan in cells. However, the administration of Mino and Sino could significantly reverse the cell damage of NPCs. It is known that the loss of type II collagen is considered to be an early indicator of IVDD, so our experiment showed that M1‐type macrophages can significantly increase the severity of IVDD disease.^24^

In the experiment, we also found that the expression of IL‐10 was increased in RAW264.7 cells co‐induced by IFN‐γ and LPS. As a matter of fact, M1‐type macrophages could also activate IL‐10 expression, but the effect is not as great as that of M2. In addition, a previous study showed that IL‐10 expression was increased in RAW264.7 cells stimulated by LPS and IFN‐γ.^25^ Moreover, the expressions of ROS, MDA and SOD were upregulated in LPS‐induced NPCs, which may be due to the fact that LPS alone can passively activate the antioxidative stress mechanism of mitochondria.

Growth factors, such as bFGF and TGF‐β1, macrophages and mast cells might play a key role in the repair of the injured annulus fibrosus and subsequent IVDD.^26^ Therefore, future studies will focus on connecting relevant factors in macrophages with the pathogenesis of IVDD, and deeply exploring the relationship between the polarization of macrophages and IVDD.

There are also some limitations in our paper. We only conducted discussions in cell experiments, and did not further verify the findings in animals. The findings discovered in this study will be verified in animals in future experiments. In addition, we have not further discussed the regulatory mechanism of macrophages, which should also need to be further investigated.

Overall, we conclude that M1 polarization of macrophages could promote LPS‐induced inflammation and damage of NPCs. Our article might provide a theoretical basis for the treatment of IVDD.

## AUTHOR CONTRIBUTIONS

Dong Chen and Zhidong Liu conceived and designed the study. Xuefeng Hou and Yucheng Shen performed the experiments. Minli Sun and Bing Zhang analyzed the experimental data. Dong Chen, Jiuming Dai and Zhidong Liu wrote and revised the manuscript. All authors have read and approved the final manuscript.

## CONFLICT OF INTEREST

The authors declare no conflict of interest.

## ETHICS STATEMENT

Ethics approval for the use of primary human cells was obtained from Binhai County People's Hospital.

## Data Availability

The analyzed data sets generated during the present study are available from the corresponding author on reasonable request.
